# Supplementary material for: ESCRT-III-driven piecemeal micro-ER-phagy remodels the ER during recovery from ER stress
Source: Nat Commun. 2019 Nov 7;10:5058. doi: 10.1038/s41467-019-12991-z (PMC6838186; doi:10.1038/s41467-019-12991-z)

# ESCRT-III-driven piecemeal *micro*-ER-phagy remodels the ER during recovery from ER stress

**Marisa Loi et al.**

# Supplementary Information

## Supplementary Figure 1

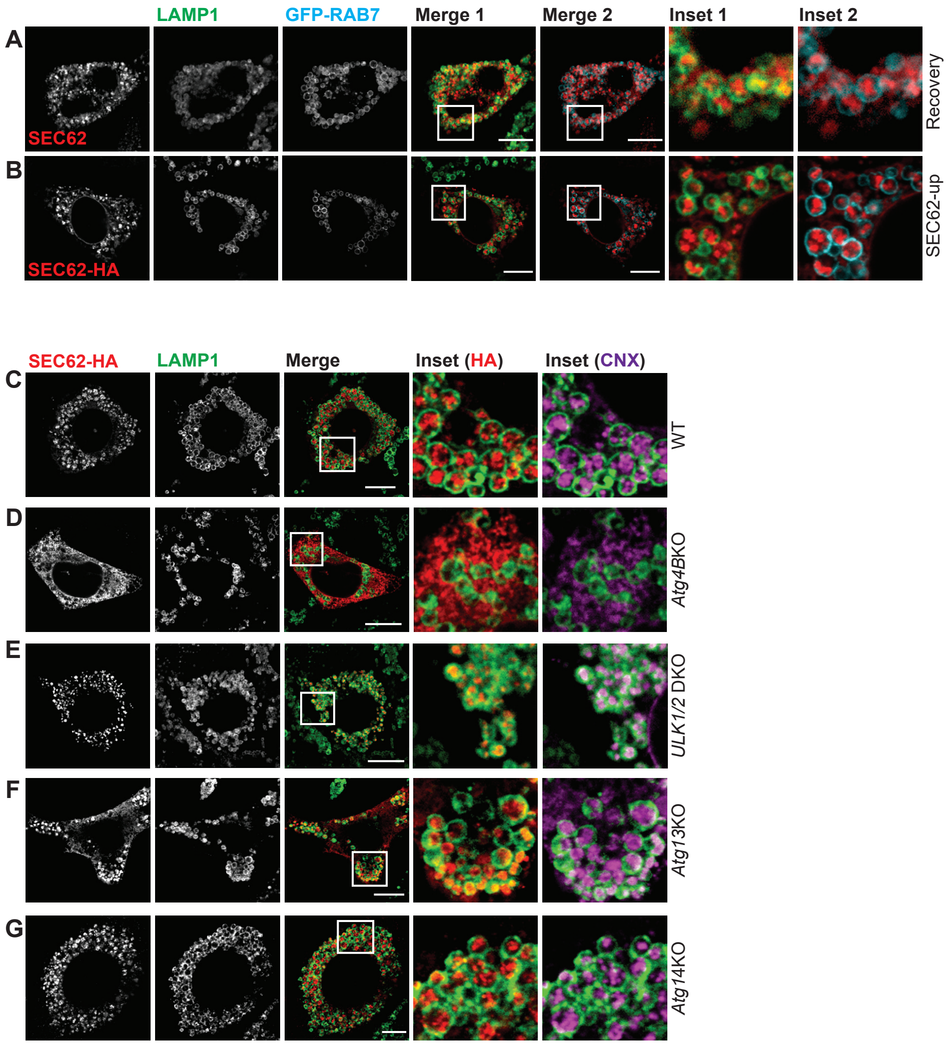


**Supplementary Figure 1. A** Endogenous SEC62 is delivered inside LAMP1 and GFP-RAB7 positive EL during recovery from CPA-induced ER stress in WT MEF exposed to 50 nM BafA1 for 12 h. **B** same as **A** when recov-ER-phagy is mimicked on overexpression of recombinant SEC62-HA in WT MEF exposed to BafA1 for 12 h. **C-G** Recov-ER-phagy mimicked on SEC62-HA overexpression in WT MEF and in *Atg4*B-, *Ulk1/2*-, *Atg13*- and *Atg14*KO MEF exposed to BafA1 for 12 h. Data are representative of at least three independent experiments. Scale bars: 10 µm.

**Supplementary Figure 2**

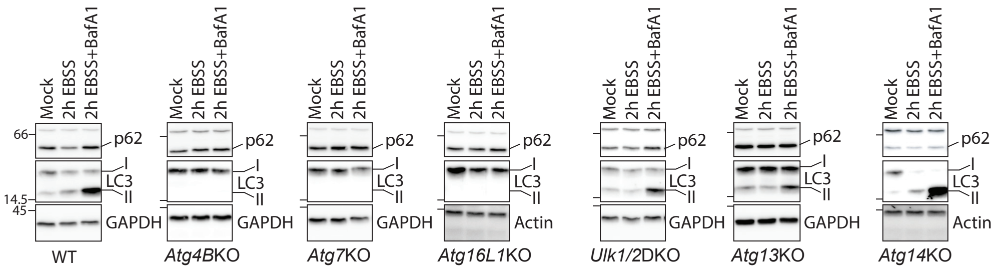


**Supplementary Figure 2.** Control of p62 turnover (upper panel) and LC3 lipidation (middle panel) in the WT MEF and in MEF KO used in this study. GAPDH and actin as loading controls. Molecular weight markers are in kDa. Data shown in this figure confirm the results published by the groups sharing these cell lines (cited in the text and in the Acknowledgements section).

**Supplementary Figure 3**

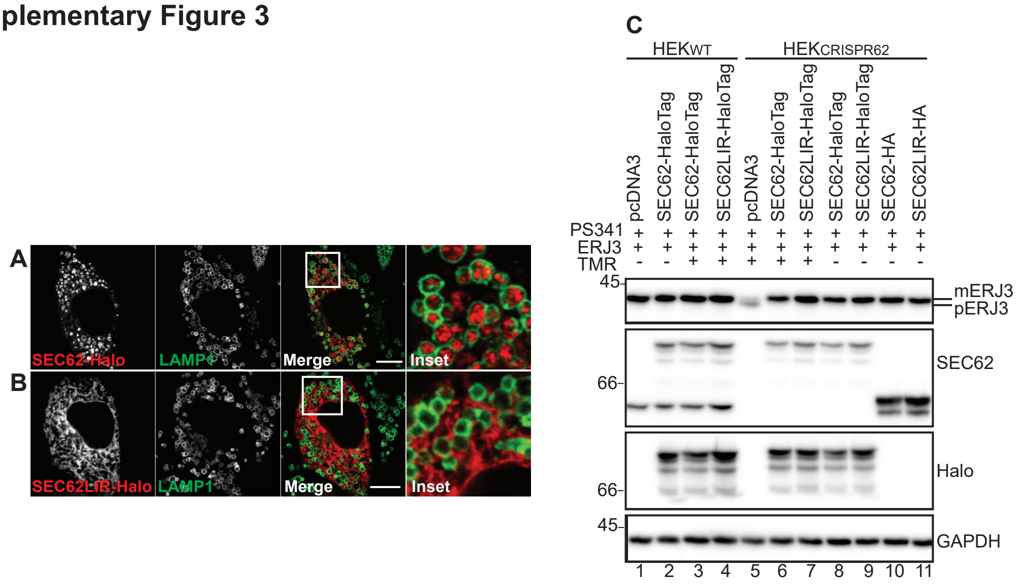


**Supplementary Figure 3. A** Delivery of SEC62-HaloTag within LAMP1 positive EL in WT MEF exposed to 50 nM BafA1 for 12 h. **B** SEC62LIR-HaloTag is not delivered within the EL. Data are representative of at least three independent experiments. Scale bars: 10 µm. **C** Translocation assay showing compromised ERj3 translocation (lane 5) in HEK CRISPR62 cells and confirming the functionality of SEC62-HaloTag and SEC62LIR-HaloTag in re-establishing ERj3 translocation (lane 6-9) in CRISPR62 cells. Molecular weight markers in WB are in kDa. WB is representative of at least three independent experiments (please also refer to [7]). Mature (m)ERj3 has slower electrophoretic mobility than pre-(p)ERJ3 due to N-glycosylation that only occurs when the protein is translocated within the ER.

**Supplementary Figure 4**

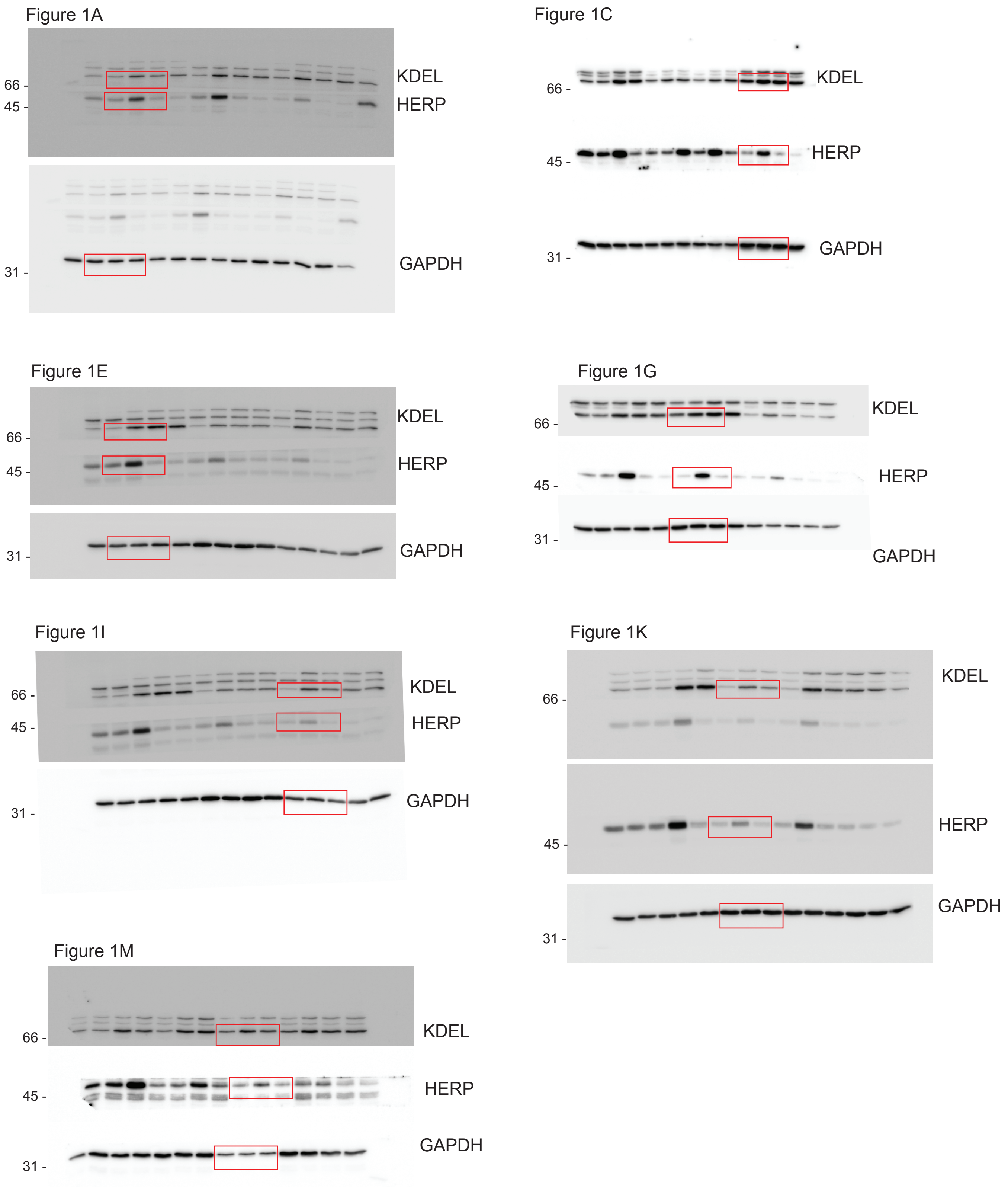


**Supplementary Figure 4 - Uncropped Western blots.**

## Supplementary Figure 4

Figure 3A

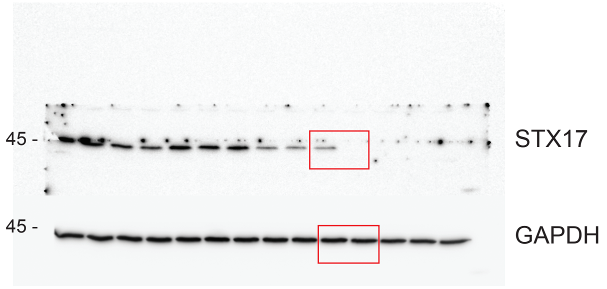


Figure 3B

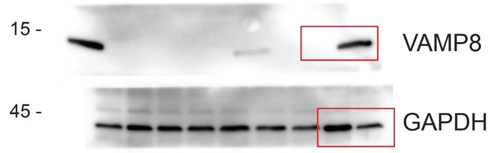


Figure 3G

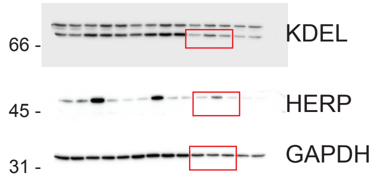


Figure 3H

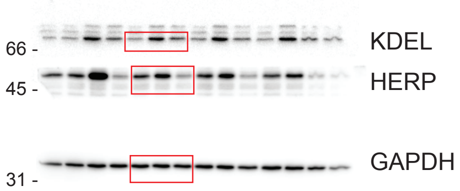

## Supplementary Figure 4

Figure 5A

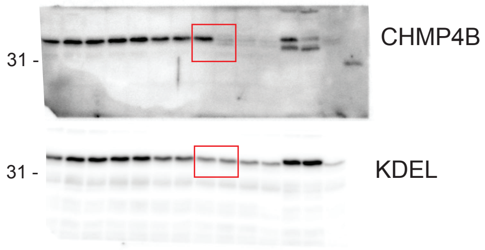


Figure 5H

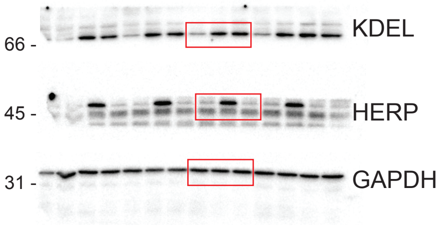

**Supplementary Figure 4**

Supplementary Figure 2

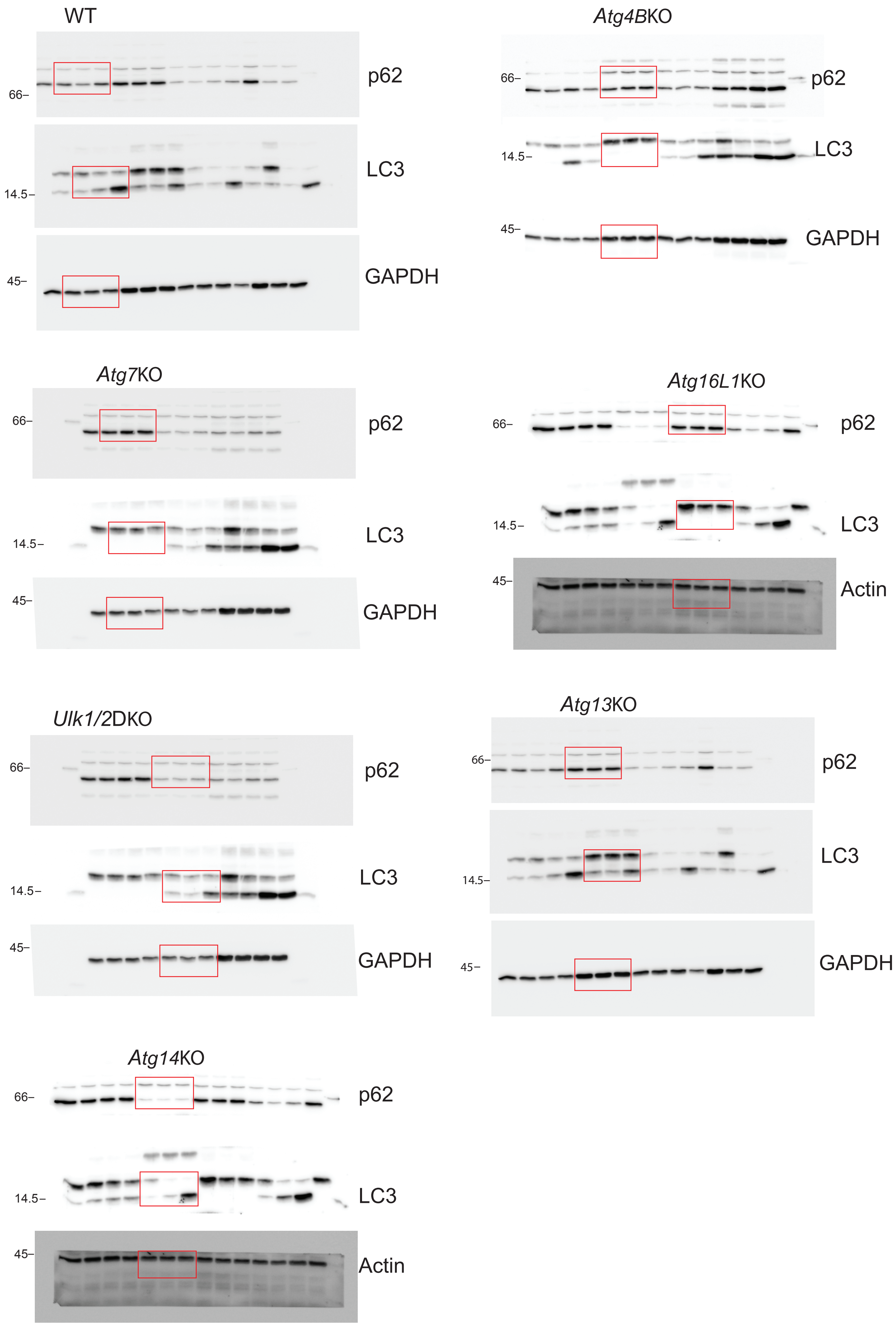

## Supplementary Figure 4

Supplementary Figure 3C

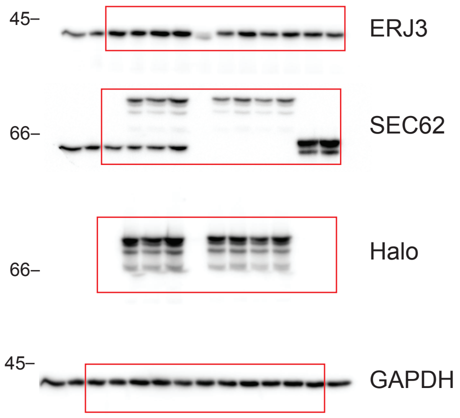

Supplement: Supplementary file 1 — Supplementary Information [file 41467_2019_12991_MOESM1_ESM.pdf]
