# Supplementary material for: ESCRT-III-driven piecemeal micro-ER-phagy remodels the ER during recovery from ER stress
Source: Nat Commun. 2019 Nov 7;10:5058. doi: 10.1038/s41467-019-12991-z (PMC6838186; doi:10.1038/s41467-019-12991-z)
Supplement: Supplementary file 5 — Reporting Summary [file 41467_2019_12991_MOESM5_ESM.pdf]

nature research

Corresponding author(s): Dr. Maurizio Molinari
Last updated by author(s): Oct 2, 2019

Reporting Summary

Nature Research wishes to improve the reproducibility of the work that we publish. This form provides structure for consistency and transparency in reporting. For further information on Nature Research policies, see Authors & Referees and the Editorial Policy Checklist.

Statistics

For all statistical analyses, confirm that the following items are present in the figure legend, table legend, main text, or Methods section.

n/a	Confirmed	
☐	☒	The exact sample size (*n*) for each experimental group/condition, given as a discrete number and unit of measurement
☐	☒	A statement on whether measurements were taken from distinct samples or whether the same sample was measured repeatedly
☐	☒	The statistical test(s) used AND whether they are one- or two-sided<br>*Only common tests should be described solely by name; describe more complex techniques in the Methods section.*
☒	☐	A description of all covariates tested
☐	☒	A description of any assumptions or corrections, such as tests of normality and adjustment for multiple comparisons
☐	☒	A full description of the statistical parameters including central tendency (e.g. means) or other basic estimates (e.g. regression coefficient) AND variation (e.g. standard deviation) or associated estimates of uncertainty (e.g. confidence intervals)
☐	☒	For null hypothesis testing, the test statistic (e.g. *F*, *t*, *r*) with confidence intervals, effect sizes, degrees of freedom and *P* value noted<br>*Give P values as exact values whenever suitable.*
☒	☐	For Bayesian analysis, information on the choice of priors and Markov chain Monte Carlo settings
☒	☐	For hierarchical and complex designs, identification of the appropriate level for tests and full reporting of outcomes
☒	☐	Estimates of effect sizes (e.g. Cohen's *d*, Pearson's *r*), indicating how they were calculated

Our web collection on statistics for biologists contains articles on many of the points above.

Software and code

Policy information about availability of computer code

Data collection	Confocal images were acquired using a Leica SP5 confocal system with LAS AF 2.7.3.9723 software.<br>3D deconvolutions were performed with Autoquant X3.1.3<br>TEM images acquired using a Talos L120C with 4k×4K Ceta CMOS camera by TIA version 4.22 software.<br>TEM Tomography on a Talos L120C with 4k×4K Ceta CMOS camera by Tomography version 4.8 software<br>TEM images acquired using a Zeiss LEO 512 TEM by a 2kx2K slow-scan Proscan camera controlled by EsivisionPro ver. 3.2 software.<br>TEM tomography using a Tecnai G2 20 TEM with an Eagle 2k×2k CCD camera using Xplore3D ver. 3.0 software.<br>Immunoblots were acquired with the ImageQuant LAS 4000 system by version 1.2 or with the Amersham Imager 680 system version 2.0.
Data analysis	IMOD ver. 4.7-4.9<br>Microscope Image Browser ver. 2.50-2.511<br>ImageJ/Fiji 1.52p<br>Statistical analyses were performed using GraphPad Prism 7 software. Immunoblots were analyzed using the Multi Gauge Analysis Tool V3.0.

For manuscripts utilizing custom algorithms or software that are central to the research but not yet described in published literature, software must be made available to editors/reviewers. We strongly encourage code deposition in a community repository (e.g. GitHub). See the Nature Research guidelines for submitting code & software for further information.

Data

Policy information about availability of data

All manuscripts must include a data availability statement. This statement should provide the following information, where applicable:

- Accession codes, unique identifiers, or web links for publicly available datasets
- A list of figures that have associated raw data
- A description of any restrictions on data availability

The source data that support the findings of this study are available from the corresponding author upon request.

Field-specific reporting

Please select the one below that is the best fit for your research. If you are not sure, read the appropriate sections before making your selection.

☒ Life sciences ☐ Behavioural & social sciences ☐ Ecological, evolutionary & environmental sciences

For a reference copy of the document with all sections, see nature.com/documents/nr-reporting-summary-flat.pdf

Life sciences study design

All studies must disclose on these points even when the disclosure is negative.

Sample size	Experiments were performed at least two times and in many cases repeated with different techniques to confirm the data. Sample size (e.g., number of cells analyzed) is specified for each experiment in the figure legend. Also, the number of independent experiments (biological replicates) is indicated in each figure legend.
Data exclusions	No data were excluded from the analyses.
Replication	As specified in the figure legend, each experiment was performed and reproduced with similar results at least twice, and up to more than 10 times (biological replicates).
Randomization	No randomization was performed as this is not common for western blot data. Imaging acquisition/analysis was performed by at least two different scientists.
Blinding	The investigators were not blinded during the experiments. Phenotypes were assessed by WB quantified with Multi Gauge Analysis Tool (blinding not required). Unbiased imaging data collection/analyses were performed by at least two different scientists with similar results. All conclusions in the study were made based on the statistical analyses.

Reporting for specific materials, systems and methods

We require information from authors about some types of materials, experimental systems and methods used in many studies. Here, indicate whether each material, system or method listed is relevant to your study. If you are not sure if a list item applies to your research, read the appropriate section before selecting a response.

Materials & experimental systems

n/a	Involved in the study
☐	☒ Antibodies
☐	☒ Eukaryotic cell lines
☒	☐ Palaeontology
☒	☐ Animals and other organisms
☒	☐ Human research participants
☒	☐ Clinical data

Methods

n/a	Involved in the study
☒	☐ ChIP-seq
☒	☐ Flow cytometry
☒	☐ MRI-based neuroimaging

Antibodies

Antibodies used	SEC62 gift from R. Zimmermann<br>ERj3 gift from R. Zimmermann<br>CNX gift from A. Helenius<br>CHMP4B gift from H. Stenmark<br>KDEL Stressgen ADI-SPA-827 02231116<br>Herp Chondrex 7039 HT2<br>HA Sigma H6908 031M4849<br>HA-probe Santa Cruz F7 sc-7392

	Lamp1 DSHB 1D4B<br>GAPDH Merck Millipore MAB374 6c5<br>p62 MBL PM045 017<br>STX17 Sigma HPA001204 C91833<br>LC3B Novus NB100-2220 BH<br>LC3B Sigma L7543 046M4787V<br>Vamp8 Abcam ab76021<br>Actin Santa Cruz sc-1616 I1<br>Halo Promega G921A<br><br>rabbit IgG light chain (HRP-conjugated) Jackson Immunoresearch 211-032-171 5A6-1D10 103988<br>goat IgG (HRP-conjugated) Santa Cruz sc-2020 I0805<br>mouse IgG (H+L) (HRP-conjugated) SouthernBiotech 1031-05<br><br>Mouse (Alexa Fluor® 488 conjugated) Jackson Immunoresearch 115-545-166 124083<br>Mouse (Alexa Fluor® 568 conjugated) ThermoFisher A-11031 1736975<br>Rat (Alexa Fluor® 647 conjugated) ThermoFisher A-21247 2068255<br>Rat (Alexa Fluor® 568 conjugated) ThermoFisher A-11077 1692966<br>Rabbit (Alexa Fluor® 488 conjugated) Invitrogen A-11008 51385A<br>Rabbit (Alexa Fluor® 647 conjugated) Jackson Immunoresearch 111-605-144 107714<br>Rabbit (Alexa Fluor® 568 conjugated)ThermoFisher A-11036<br>Rabbit (Brilliant Violet 421) BioLegend 406410
Validation	Antibodies used in this study were obtained from commercial sources and validated according manufacturer's instruction.<br>Non commercial antibodies for SEC62, ERj3 and CNX were validated in previous work (Fumagalli et al, NCB 2016).<br>STX17 and VAMP8 antibodies were validated in Fregno et al. EMBO J 2018 and again here by CRISPR (Fig. 3A and B, respectively).<br>CHMP4B antibody was described previously (Sagona et al, NCB 2010) and was validated here by siRNA (Fig5A).

Eukaryotic cell lines

Policy information about cell lines

Cell line source(s)	ATG7 (WT and KO) MEF are kind gifts of M. Komatsu, ATG4BKO of G. Marino, ULK1/2DKO of S. Tooze, ATG13KO of F. Reggiori, ATG14 and ATG16L1 of T. Saitoh. MEFs CRISPR WT, STX17 and VAMP8 were generated in the lab as described previously (Fregno et al, 2018). HEK293 cells from ATCC. CRISPR SEC62 HEK cells (Fumagalli et al 2016 and this work), CRISPR STX17 and CRISPR VAMP8 MEF (Fregno et al 2018 and this work) were generated and characterized in our lab.
Authentication	The identity of the various autophagy-deficient cell lines was checked by functional analyses (p62 turnover and LC3 lipidation on autophagy induction) as shown in Extended Data Fig. 2.
Mycoplasma contamination	Cells were regularly tested to be negativ for mycoplasma infection.
Commonly misidentified lines<br>(See ICLAC register)	No commonly misidentified cell lines were used in this study.
